# GalNAc-Transferases in Cancer

**DOI:** 10.3390/biomedicines14010005

**Published:** 2025-12-19

**Authors:** Shruthi C. Iyer, Dinesh Kumar Srinivasan, Rajeev Parameswaran

**Affiliations:** 1 Yong Loo Lin School of Medicine, National University of Singapore, Singapore 117597, Singapore; 2 Department of Anatomy, Yong Loo Lin School of Medicine, National University of Singapore, Singapore 117594, Singapore; dineshkumar@nus.edu.sg; 3 Department of Surgery, National University Hospital, Singapore 119074, Singapore

**Keywords:** GALNT, biomarker, cancer, metastasis, prognosis, chemotherapy

## Abstract

**Background/Objectives**: The polypeptide N-acetylgalactosaminyltransferase (GALNT) family initiates mucin-type *O*-glycosylation, a post-translational modification that plays a pivotal role in cellular signaling, adhesion, and immune evasion. Dysregulated GALNT expression has been increasingly implicated in carcinogenesis. **Methods**: We reviewed the literature on the expression, function, and clinical relevance of GALNT isoforms across various cancers, with a focus on their mechanistic roles, biomarker potential, and therapeutic implications. **Results**: Aberrant GALNT expression is observed in numerous malignancies, including breast, colorectal, gastric, lung, ovarian, and hepatocellular carcinomas. Isoforms such as GALNT1, -T2, -T3, and -T14 contribute to tumorigenesis by modulating the glycosylation of mucins such as Mucin-1 (MUC1), epithelial growth factor receptors (EGFR), and other signaling proteins. These alterations promote cancer cell proliferation, metastasis, epithelial–mesenchymal transition (EMT), and chemoresistance. Deranged GALNT expression is frequently associated with poor prognosis, and certain GALNT genotypes predict treatment response. However, functional redundancy among isoforms poses challenges for selective targeting. **Conclusions**: Despite their strong potential as modulators of cancer progression, GALNTs face substantial limitations in terms of substrate identification, mechanistic clarity, immune relevance, and therapeutic tractability. Overcoming these challenges requires advanced glycoproteomics, development of isoform-specific tools, and integrated studies across cancer and immunology to fully harness GALNT biology for clinical benefit.

## 1. Introduction

Protein glycosylation is an essential post-translational modification step in eukaryotes, with many metabolically active substances being glycosylated compounds (glycans). Numerous protein glycosylation types exist in animals, plants, and microorganisms [1]. Mucin-type *O*-glycosylation is one such type and occurs when glycans are attached to serine (Ser) and threonine (Thr) amino acid residues via *O*-linked N-acetylgalactosamine (GalNAc) [2]. Biologically, mucin-type *O*-glycosylation plays a critical role in maintaining epithelial barrier function, modulating immune responses, and regulating protein secretion and turnover. The polypeptide N-acetylgalactosaminyltransferases (GALNTs) thus contribute to proteome complexity and functional diversification, particularly in mucosal tissues where mucins—heavily *O*-glycosylated glycoproteins—form protective layers [2,3].

Typically, the initiation of *O*-glycosylation is mediated by only one or two genes encoding glycosyltransferase (GT) enzymes. These genes encode enzymes that attach the first *O*-glycan to the relevant amino acid residue in the protein. However, the initiation step of mucin-type *O*-linked glycan synthesis involves a large family of up to 20 homologous genes which encode numerous GALNT enzymes [2]. GALNTs are a type of GT enzyme that catalyze *O*-glycosylation to form mucin-type *O*-glycans, and this process is initiated in the Golgi apparatus once most of the protein folding events have been completed [4]. This contrasts with N-glycosylation and other types of *O*-glycosylation, which is initiated in the endoplasmic reticulum (ER). The larger number of enzymes initiating mucin-type *O*-glycan synthesis and its occurrence in the Golgi apparatus rather than in the ER make GALNTs unique compared to other types of protein glycosylation enzymes. This is due to their greater potential for differential regulation in cells and tissues [4].

Clinically, aberrant GALNT activity is associated with several pathological conditions. Malfunction of GALNTs is responsible for altered glycosylation and a truncated mucin-type *O*-glycan structure, which have been associated with increased metastatic potential and poor prognosis in cancer [5,6,7]. In cancer, altered expression of specific GALNTs (e.g., GALNT3, T6, etc.) leads to the synthesis of truncated or atypical *O*-glycans, such as the Tn and sialyl-Tn antigens, which are hallmarks of tumor progression and metastasis. This review aims to explore the role of GALNTs in cancer, their underlying molecular mechanisms, and their potential as prognostic markers for various cancers.

### 1.1. Structure of GALNTs

GALNTs, like other Golgi glycosyltransferases, have a type II transmembrane topology. They have a short N-terminal cytoplasmic domain, a single hydrophobic transmembrane domain, and a large luminal/extracellular C-terminus [8]. The short cytoplasmic domain of all GALNTs contains a basic sequence of amino acids that may be involved in interaction with peripheral Golgi membrane protein tethering complexes [8]. The luminal domain consists of a juxtamembrane stem region and a catalytic region. The additional ricin-like lectin domain of GALNTs at the C-terminus makes them unique compared to other eukaryotic GTs [9]. The juxtamembrane stem regions are variable in length across the different isoforms of GALNTs (ranging from around 90 amino acids in GALNT1, -T13, and -T16 to 470 amino acids in GALNT5) [8].

**Figure 1:**
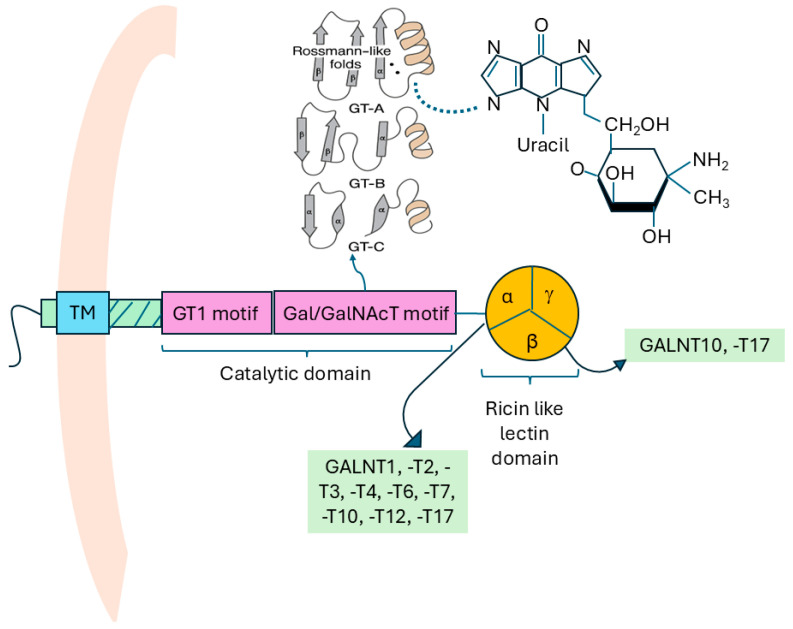
The domain architecture of GALNT showing linkage between GT-A motif and uracil ring of the UDP-GALNT molecule. The various GALNT enzymes that bind specifically to the alpha and beta domains of the ricin like lectin are highlighted in the green box (as indicated by the arrows). Abbreviations: GALNT—polypeptide N-acetylgalactosaminyltransferase; GT-A—glycosyltransferase-A; UDP—uridine diphosphate.

The GALNT catalytic domains are about 230 amino acids long and adopt three main three-dimensional (3D) fold patterns called the GT-A, GT-B, and GT-C, respectively [4,10]. GT-A enzymes are metal-ion-dependent, using a metal ion cofactor (primarily Mn^2+^) at the active site to catalyze the reaction. The GT-A structural motif, a specific 3D fold common in glycosyltransferases, made up of two tightly interacting β–α–β Rossmann-like folds, contains amino acids that interact with the uracil ring of the Uridine diphosphate N-acetylgalactosamine (UDP-GalNAc) molecule, which is the sugar donor in the enzymatic reaction (as shown in Figure 1) [4,11]. In contrast, GT-B enzymes possess two distinct domains and are metal-ion-independent, thereby catalyzing reactions even without a metal ion cofactor at the active site. More recently, a third folding pattern called GT-C was identified [12]. GALNTs, like other GTs, may be phosphorylated either at their cytosolic or luminal domains, and this provides insight into how GALNT activities could be regulated by phosphorylation. In a similar manner, the GALNT activation (GALA) pathway drives the re-localization of GALNTs from the Golgi to the ER, partly through negative regulation of Extracellular Signal-Regulated Kinase 8 (ERK8). This activation of the GALA pathway leads to the accumulation of truncated *O*-glycans in cancer cells, which in turn enhances tumor cell migration, invasion, and metastatic behavior [13,14,15,16].

### 1.2. Types of GALNTs and the Human GALNT Gene Family

GALNTs are members of the GT27 family in the CAZy glycosyltransferase classification (http://www.cazy.org (accessed on 3 August 2024)). In humans, 20 GALNT genes have been identified, most of which encode active polypeptide GALNTs functioning in *O*-glycosylation [4]. The genes encoding GALNTs, known as GALNT genes, have been highly conserved in sequence throughout evolution and play an essential function in eukaryotic species [4,17]. In 2012, Bennett and colleagues analyzed cladograms (phylogenetic trees based on sequence similarities) and genomic organization (with specific focus on exon-intron boundaries). They grouped the human GALNTs into seven subfamilies (namely subfamilies Ia–g, IIa, and IIb) [4]. Most of the GALNTs show enhanced expression in specific tissues, as shown in Table 1. The differential expression of the GALNT genes across various tissues and developmental stages contributes to the functional diversity of glycosylation patterns observed in human proteins [13].

**Figure 2:**
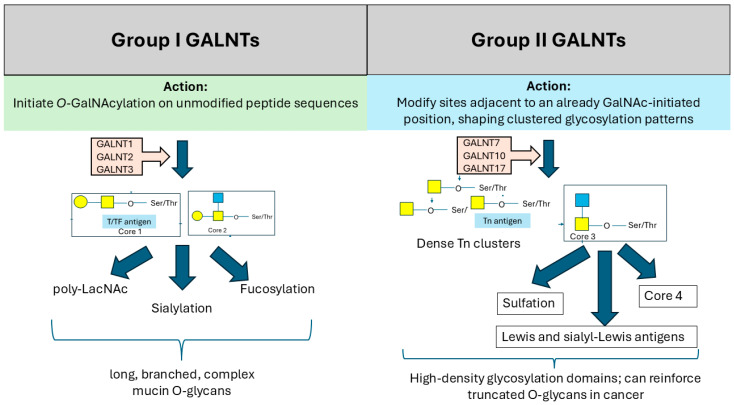
Differences in action of group I and II GALNTs. Yellow square = GalNac; blue square = GlcNac; yellow circle = galactose. Abbreviations: GALNT—polypeptide N-acetylgalactosaminyltransferase; Tn—Thomsen-nouveau; Ser—serine; Thr—threonine.

The GALNT gene family is organized into two main groups based on their structural characteristics and substrate specificity. Group I GALNT genes, such as GALNT1, GALNT2, and GALNT3, are primarily responsible for synthesizing the Tn antigen, a precursor of more complex *O*-glycans [13]. In contrast, group II gene members, such as GALNT4, GALNT5, and GALNT6, exhibit broader substrate specificity and are involved in generating more extended *O*-glycan structures (Figure 2) [13].

### 1.3. Substrate Specificities of GALNTs

The substrate specificities of GALNTs are fundamental in defining the structure and function of mucin-type *O*-glycans in diverse biological contexts. These enzymes catalyze the initial transfer of GALNT to serine or threonine residues on polypeptide chains, initiating *O*-glycosylation. Individual GALNT isoforms exhibit distinct preferences for peptide sequences and post-translational modifications, thereby contributing to the complexity and specificity of *O*-glycan patterns. Certain isoforms preferentially act on peptide motifs enriched in proline or other bulky amino acids, which can modulate both enzymatic activity and glycan structure [18,19]. Importantly, the large family of GALNT isoenzymes acts in a coordinated fashion. Some isoforms initiate *O*-glycosylation at specific sites, while others act subsequently to glycosylate adjacent residues, thereby promoting glycan extension and density [9,20]. Additionally, some isoforms selectively glycosylate sites already modified by other glycosyltransferases, whereas others display broader substrate tolerance, enabling the modification of a wider range of peptide sequences [19]. Some examples of isoform specific expression affecting substrate selection and glycan function are listed in Table 2 [21,22,23,24,25,26,27,28,29,30,31].

### 1.4. Expression of GALNTs

The expression of GALNTs is tightly regulated and shifts with cell type, differentiation status, and maturation stage. The expression also varies significantly between normal and cancerous tissues. In noncancerous states, specific isoforms of GALNTs are expressed in a controlled manner, contributing to the synthesis of non-truncated mucin-type *O*-glycans, usually capped with sialic acid. For example, correct glycosylation mediated by GALNTs is important in the functioning of many peptide hormones, lipid regulation, and bone functions [32,33,34]. In contrast, cancerous tissues exhibit altered expression levels of GALNTs, leading to aberrant glycosylation patterns that can promote tumor progression and metastasis [35]. The mechanisms through which GALNTs play a role in cancer are described in Table 2. The truncated glycoforms seen in cancer states are possibly the result of various mechanisms involving GALNTs, some of which include core-1 β1-3galactosyltransferase-specific chaperone 1 (COSMC) inactivation, through mutations or hypermethylation [36,37,38], relocation of GALNTs to the ER instead of Golgi apparatus [14,39], structural/topological changes in glycosylation enzymes [39,40], and impaired stability of E-cadherins, thereby giving rise to altered cell adhesion molecules [41].

### 1.5. Role of Golgi Apparatus in GALNT Activity

The Golgi apparatus plays a crucial role in the function and localization of GALNTs. The Golgi is a key organelle of the secretory pathway and consists of stacks of cisternae, which receives the proteins and lipids synthesized in the ER to further process them [42]. The cisternae are interconnected to form Golgi ribbons, which are located near the centromere. The stack is sandwiched at the entrance by the membrane structures called the cis-Golgi network (CGN) and vesicular tubular clusters (VTCs), and at the exit by the trans-Golgi network (TGN) [42,43,44]. The TGN is the major site which sorts proteins to distinct cellular locations.

Inside the Golgi, GALNTs catalyze the addition of GalNAc to the hydroxyl groups of serine and threonine residues on target proteins, initiating *O*-linked glycosylation to form various glycans (Figure 2). Their precise localization within Golgi sub-compartments is essential for determining where, when, and how extensively *O*-glycans are added [33,45]. Enzymes involved in glycosylation are generally arranged according to their function: early-acting enzymes are found in the cis-Golgi, while late-acting ones are enriched in the trans-Golgi [17,46,47,48]. However, not all glycosylation enzymes follow a strict cis-to-trans localization. For example, GALNTs involved in the initiation of *O*-glycosylation—specifically GALNT1, -T2, and -T3—are distributed across different regions of the Golgi [47]. Furthermore, whilst GALNT1 is evenly distributed throughout the stack, GALNT2 localizes mainly to the trans-Golgi, and GALNT3 is enriched in the medial Golgi.

**Figure 3:**
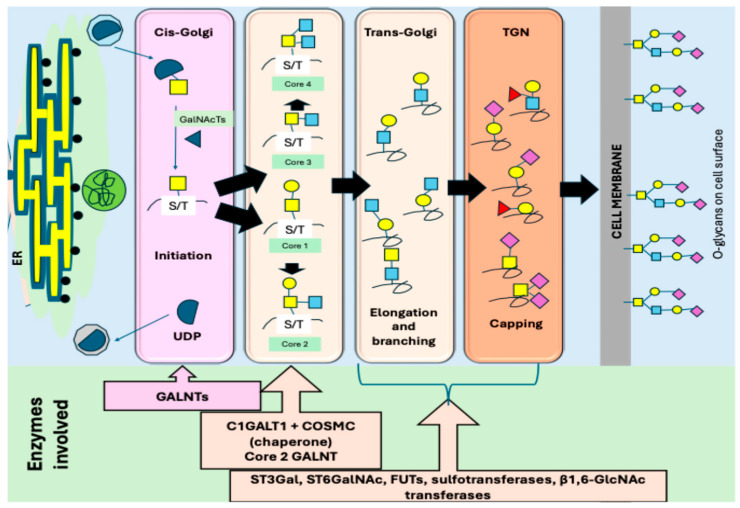
The synthesis and movement of the *O*-glycans along the compartments of the Golgi apparatus to be expressed on the cell surface. The key to glycans is as follows: yellow square = GalNac; blue square = GlcNac; yellow circle = galactose; purple diamond = sialic acid; red triangle = fucose; blue triangle arrow to shown GALNT acting on the reaction step. The dark bold arrows indicate movement of glycans from Golgi compartment to another. The small arrows in the cis-Golgi show the pathway of UDP-GalNAc to form Tn epitope. Abbreviations: UDP—uridine diphosphate; GALNT—polypeptide N-acetylgalactosaminlytransferase; TGN—trans-Golgi network; C1GALT1—core 1 synthase, glycoprotein-N-acetylgalactosamine 3-beta-galactosyltransferase 1; COSMC—Core-1 β1-3galactosyltransferase-specific chaperone 1; ST3Gal—ST3 beta-galactoside alpha-2,3-sialyltransferase 1; FUT—Fucosyltransferase; GlcNAc—N-Acetylglucosamine.

When the GALNTs shift to later Golgi compartments, initiation becomes restricted due to increased protein folding and prior modifications, altering glycan site occupancy and density [49]. The location of GALNTs also determines the timing of competition between initiation and downstream enzymes such as C1GALT1, GCNTs, and sialyltransferases, shaping core structure formation and glycan elongation [33,49,50]. Signal-regulated GALNT relocalization (e.g., via EGFR–ERK) can enhance global initiation, modify receptor glycosylation, and alter cell signaling, a phenomenon frequently observed in cancer, which is discussed in a later section. Thus, GALNT spatial organization governs the fidelity, extent, and biological impact of *O*-glycan biosynthesis. Mislocalization due to trafficking defects, pH disruption, or Golgi stress contributes to truncated Tn/STn antigens and disease-associated glycosylation abnormalities [49,51,52]. The Tn glycan is extended by the addition of more sugars to produce a mature *O*-GalNAc glycan, each with one of four core molecules (Figure 3).

The synthesis and movement of the *O*-glycans are initiated in the cis-Golgi bodies by GALNTs in normal conditions. However, there is an alternative pathway called the GALA pathway, whereby the GALNTs are relocated from the Golgi to the ER, enabling the glycosylation of substrates in the ER, a site where glycosylation typically does not occur [53]. In the GALA system, external stimuli (e.g., growth factors) activate Src, a non-receptor tyrosine kinase, which is essential for initiating the relocation of GALNTs, thereby triggering signaling cascades. Src activation enhances activity of the Coat Protein I (COPI) vesicular transport system, enabling retrograde transport of GALNTs to the ER. This step is GALNT-specific; other Golgi enzymes are not significantly affected. ERK8, a serine/threonine-specific protein kinase, inhibits GALNT relocation. When ERK8 is downregulated or inactivated, GALNTs more easily translocate to the ER. In the ER, they gain access to immature, newly synthesized proteins, allowing for early glycosylation and modification of proteins that would otherwise not be glycosylated. This is an atypical and inducible pathway of *O*-glycosylation, usually seen in cancer states, which leads to the abundant formation of the Tn antigen [39,54].

## 2. Role of GALNTs in Cancers

Aberrant expression and mutations in GALNTs can lead to cancer-specific glycosylation patterns, impacting tumor proliferation, induction of EMT, metastasis, immune evasion, and drug resistance [55,56,57]. Many of the functions of receptors such as EGFR, Notch, integrins, etc., rely on proper glycosylation mediated by GALNTs and influence the various cancer signaling pathways. This has been shown in many human cancers, which will be discussed below.

### 2.1. EGFR-Mediated Pathway

Epithelial growth factor (EGF) and EGF receptor activation are involved in the development and progression of many cancers. Ligand binding of EGFR results in phosphorylation and dimerization, with resultant downstream cascade activation of the various signaling pathways, resulting in increased proliferation, migration, and invasion of cancer cells. Glycosylation of EGFR by GALNTs alters receptor conformation, stability, localization, or internalization [23]. GALNT1 overexpression is associated with increased cell migration and invasion. Using hepatocellular carcinoma (HCC) cell lines, Huang et al. showed that the knockdown of GALNT1 reduced EGF-induced EGFR phosphorylation and enhanced EGFR degradation [23]. Overexpression of GALNT2 suppresses EGF-induced EGFR activation and promotes EGFR degradation. Wu et al. showed that the *O*-glycosylation status of EGFR was altered by GALNT2 using immunoprecipitation techniques with Vicia Villosa Lectin (VVA-lectin), and specifically, GALNT2 knockdown resulted in higher EGFR phosphorylation and downstream signaling [21]. Similarly, using gastric cancer cells, knockdown of GALNT2 decreased Tn epitope expression on EGFR and increased EGFR/Akt downstream signaling, with a resultant increase in malignant behavior [58]. Glycosylation of EGFR by GALNT5 promotes ligand binding and stability of EGF, leading to the activation of the AKT/ERK axis, promoting malignant behavior in cholangiocarcinoma cell lines; however, the precise *O*-glycosite residues on EGFR modified by GALNT5 have not been mapped [59].

GALNT3 also acts as a modulator/suppressor of EGFR activity via *O*-glycosylation of EGFR (or its co-receptors). In pancreatic cancer cell lines, reduced GALNT3 expression was associated with increased phosphorylation of EGFR/ErbB family receptors and increased aggressiveness of poorly differentiated cancer cells [60]. The knockdown of GALNT3 resulted in increased Tn antigen expression on EGFR and human epidermal growth factor receptor 2 (Her2) proteins (i.e., truncated *O*-glycans) and increased receptor activation. Using pull-down assays with VVA-lectin in chemoresistant ovarian cancer, Li et al. showed GALNT14 to be involved with the EGFR/mTOR pathway that contributed to cisplatin resistance [26]. This study showed that the downregulation of GALNT14 significantly inhibits *O*-GALNTylation of EGFR and promotes apoptosis and ferroptosis of ovarian cancer cells. Besides direct glycosylation of EGFR, GALNTs may indirectly alter the glycosylation of receptors/co-receptors or integrins that modulate EGFR signaling [55]. The differential effects of GALNTs on EGFR signaling likely reflect site-specific *O*-glycosylation patterns, with distinct substrate specificities.

### 2.2. TGF-β Signaling Pathway and Epithelial–Mesenchymal Transition (EMT)

Transforming growth factor beta (TGF-β) plays dual roles in cancer, acting as a tumor suppressor during early stages of cancer, but as a tumor promoter of invasion and metastasis in late stages [61]. TGF-β receptor activation triggers both SMAD-dependent and SMAD-independent pathways that drive EMT, immune evasion, and fibrosis [62]. EMT is characterized by the coordinated loss of epithelial junction proteins, such as E-cadherin, and the acquisition of mesenchymal markers, including vimentin [63]. During EMT, epithelial cells lose their polarity and intercellular adhesion, acquire mesenchymal traits, and become motile and invasive [64].

*O*-GALNTylation by GALNT enzymes can amplify or dampen TGF-β signaling, reshaping cellular adhesion and migration [56]. GALNT4 acts as a tumor-suppressive modulator of TGF-β signaling, specifically by modifying the TGF-β type II receptor (TβRII) and restraining EMT, migration, and metastasis, as shown by Wu et al. in breast cancer cells [65]. The authors showed that a GALNT moiety attached to Ser31 on TβRII by GALNT4 causes steric hindrance, leading to the decreased dimerization of TβRII. This, in effect, decreases SMAD2/3 phosphorylation, diminishes the transcription of EMT-related genes (e.g., SNAIL, SLUG, ZEB1), and thereby helps to maintain the epithelial phenotype, with decreased invasion and migration. Knockdown of GALNT4 led to the opposite effect with the enhancement of EMT, mesenchymal markers such as SNAIL and N-cadherin, and a decrease in E-cadherin (epithelial marker) levels [65].

GALNT2 normally suppresses TGF-β pathway activation, with reduced expression leading to upregulated TGF-β ligand production, enhanced EMT, and invasion. The expression of GALNT2 can be tumor-suppressive in gastric [66] and hepatocellular carcinoma [23], whereas it can act as a tumor promoter in colorectal cancer [67] and lung cancer [68]. The differential behavior of tumor suppression versus tumor promotion is dependent on substrate activation, such as EGFR, TGF-β, etc. Liu et al., using in vivo studies, showed that knockdown of GALNT2 in SGC-7901 gastric cancer cells increased TGF-β expression, along with MMP-2, leading to increased SMAD, resulting in enhanced EMT and invasive features [66]. GALNT2 also acts independently of TGF-β by modifying *O*-glycans on AXL (via a proteasome-dependent pathway) to induce migration, invasion, and peritoneal metastasis in colorectal cancer cells [67]. Knockdown of GALNT2 (via siRNA or CRISPR) reduced colorectal cancer cell migration and invasion.

During TGF-β-induced EMT, cancer cells acquire invasive and metastatic traits in parallel with the abnormal regulation of key GALNTs [61]. In breast cancer cell lines, GALNT14 promotes the proliferation, migration, and invasion of breast cancer cells. GALNT14 expression is associated with an increase in the mRNA expression of vimentin, N-cadherin, matrix metalloproteinase-2 (MMP-2), vascular endothelial growth factor (VEGF), TGF-β, and MMP-2 activity, with opposite effects seen in GALNT14-knockdown cells [69]. Induction of oncofetal fibronectin (onfFN) with EMT by TGF-β has been shown in cancer cells, via *O*-glycan addition in the IIICS domain catalyzed by GALNT3 and/or GALNT6 [70,71]. In human prostate cancer cell lines, silencing GALNT3/GALNT6 reduced onfFN with the blunting of TGF-β-induced EMT (loss of E-cadherin, gain of N-cadherin/vimentin, and increased motility) [70]. Similarly, in breast cancer cell lines, the suppression of fibronectin expression abolished the aberrant proliferative phenotype induced by GALNT6 overexpression, indicating that the GALNT–fibronectin axis is a pivotal driver of cancer progression [29].

### 2.3. Notch Signaling and GALNTs

Notch signaling is a highly conserved juxtracrine (cell to cell) pathway, whereby a Notch receptor on a cell binds to a ligand on a neighboring cell. This triggers proteolytic cleavages of the receptor and release of the Notch intracellular domain (NICD), which then translocate to the nucleus to regulate gene expression [72,73]. The extracellular domain of Notch can undergo *O*-glycosylation, which influences ligand binding/activation [74]. This has been shown to be involved with cancer progression, including metastasis [75]. The NOTCH1 receptor can be glycosylated by GALNT11 at the EGF6 and EGF36 sites in the juxtamembrane region close to the S2 cleavage site [76]. Direct evidence linking GALNTs and Notch signaling in cancer is limited in the literature. In lung adenocarcinoma, GALNT2 promotes proliferation and progression of cancer by activation of the Notch/Hes1-PTEN-PI3K/Akt signaling pathway [77]. Inhibition of GALNT2 expression by siRNA in lung cancer cells resulted in decreased levels of Notch1/3 [78]. GALNT11 expression has been shown to be a prognostic marker for Notch-driven chronic lymphoid leukemia subtypes, particularly those with high NOTCH1/2 and JAG1/2 expression [79].

### 2.4. GALNT and Immune Evasion in Cancers

The role of GALNTs in immune evasion stems from the way glycosylation masks tumor antigens, modulates immune checkpoint ligands, and affects immune cell recruitment and activation. The enzyme GALNT6 plays an important role in immune evasion by its action of MUC1, a heavily glycosylated mucin often overexpressed in epithelial cancers [80,81,82,83]. The heavy mucin *O*-glycosylation creates a dense “glycan shield” over peptide epitopes, obscuring immune recognition (by antibodies, T cells) or interfering with antigen presentation [84]. In a study using bladder cancer cell lines, Sun et al. showed that GALNT6-driven glycosylation impaired the infiltration, cytotoxic function, and effector activity of CD8^+^ tumor-infiltrating lymphocytes (TILs) within the tumor microenvironment (TME) [85]. Mechanistically, GALNT6-mediated *O*-glycosylation upregulates the expression and function of immune checkpoint molecules such as programmed death-ligand 1 (PD-L1), Cytotoxic T-lymphocyte-associated protein 4 (CTLA-4), and Lymphocyte-activation gene 3 (LAG-3), thereby enhancing inhibitory signaling cascades that suppress anti-tumor immunity. This glycosylation-dependent modulation also attenuates immune-related signaling pathways, diminishes CD8^+^ T cell proliferation and effector function, and promotes the secretion of immunosuppressive cytokines and mediators. Collectively, these alterations foster the development of an immunosuppressive TME, facilitating tumor immune escape and supporting malignant progression.

A study using pancreatic cancer cell lines (PaTu-8988t and KPC) showed a negative association between GALNT6 expression and immune cell infiltration [86]. In this study, knockdown of GALNT6 significantly increased the sensitivity of PDAC cells to cytotoxic T lymphocytes (CTLs) and macrophages and suppressed Programmed Cell Death Ligand 1 (PD-L1) levels in both in vitro and in vivo systems. Mechanistically, GALNT6-mediated *O*-glycosylation inhibited the translocation of stimulator of interferon genes (STING) and promoted their degradation in a cyclic guanosine monophosphate-adenosine monophosphate (GMP-AMP) synthase (cGAS)-independent manner, leading to reduced expression of type I interferon (IFN-β) and the chemokines C-C motif chemokine ligand 5 (CCL5) and C-X-C motif chemokine ligand 10 (CXCL10) [80,81]. These changes suppressed the recruitment of CD8^+^ T cells and macrophages, thereby facilitating immune evasion. Furthermore, GALNT6 stabilized PD-L1 by preventing its ubiquitin–proteasome-mediated degradation, contributing to the maintenance of an immunosuppressive tumor microenvironment [82]. The authors showed that the combination of Benzyl-α-GALNT and BMS1166 and the combination of an *O*-glycosylation inhibitor with PD-1/PD-L1 immune checkpoint blockade synergistically enhanced anti-tumor immune responses and reduced the viability of the tumor cells.

An integrated multi-omics analysis has revealed that post-translational modification (PTM) networks are central drivers of colorectal cancer (CRC) progression and immune evasion [87]. The study identified the widespread dysregulation of post-translational modification (PTM) pathways, with aberrant ubiquitination sustaining Wnt/β-catenin signaling and promoting tumor growth. Notably, GALNT6-mediated glycosylation emerged as a key mechanism of immune escape by stabilizing PD-L1 and preventing CD8^+^ T cell infiltration [83,84,86]. Single-cell transcriptomics further linked GALNT6 activity to a goblet-cell-like tumor subpopulation associated with immune exclusion. A machine learning-derived 5-gene PTM Activity Signature (including GALNT6, UBE2C, and others) achieved perfect discrimination between CRC and healthy tissues and stratified patients by immune phenotype. Therapeutically, combining *O*-glycosylation inhibition with PD-1/PD-L1 blockade synergistically enhanced anti-tumor immune responses. These findings establish PTM networks—particularly GALNT6-mediated glycosylation—as pivotal regulators of tumor immune dynamics and underscore their potential as both diagnostic biomarkers and therapeutic targets in colorectal cancer.

The study by Song et al. demonstrated that GALNT14 promotes lung-specific breast cancer metastasis by enhancing tumor cell self-renewal and interactions with the lung microenvironment [88]. Clinically, high GALNT14 expression in primary tumors correlated with poor lung metastasis-free survival. GALNT14 enables breast cancer cells to overcome bone morphogenetic protein (BMP)-mediated inhibition of self-renewal in the lung by glycosylating BMP receptors, supporting stem-like behavior via SOX4 upregulation [88]. Knockdown of c-JUN resulted in the inhibition of the KRAS–PI3K pathway with decreased GALNT14 expression. In vivo data from the study also showed GALNT14′s role in the recruitment of macrophages to the site of metastases, most likely by modulating the secretion of cyto/chemokines. Furthermore, GALNT3 has been shown to exert tumor-suppressive effects by limiting angiogenesis in lung cancer through EMT–dependent pathways, underscoring its broader immunomodulatory role in cancer [89,90].

## 3. GALNTs as Prognostic Markers in Cancer

**Figure 4:**
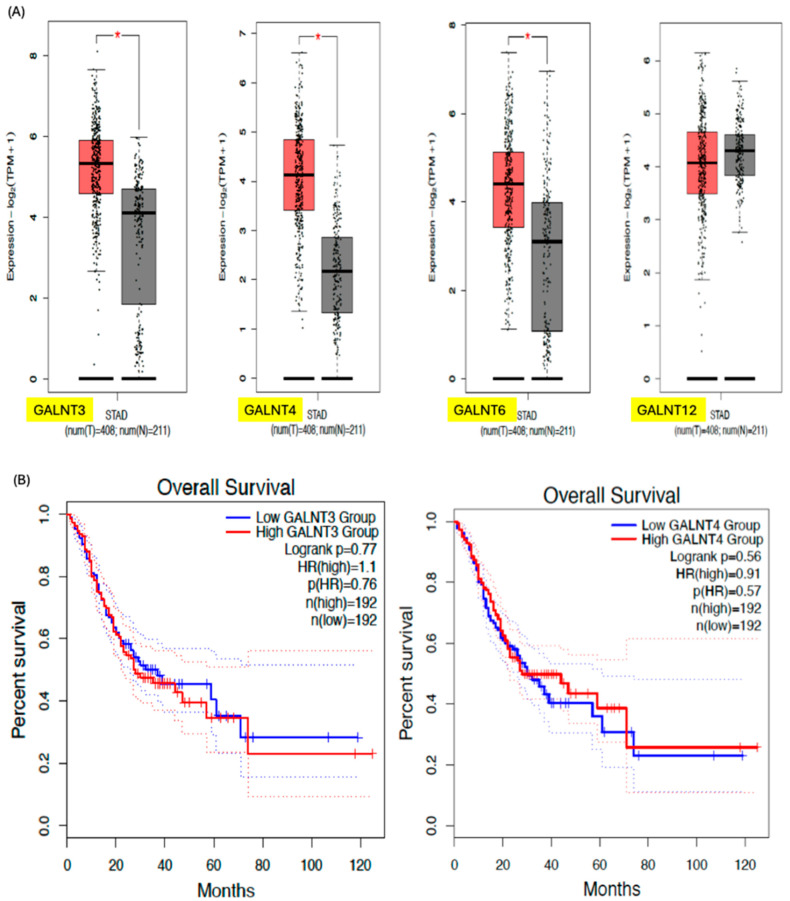
(**A**) Gene expression profile of the various GALNTs in colorectal cancer, showing higher GALNT3/4 and 6 expressions in cancer cells compared to normal cells. (**B**) Kaplan–Meier survival curve based on GALNT expressions in colorectal cancer showing no significant difference in survival based on GALNT expression. Dotted lines indicate 95% confidence interval. The figures are extrapolated from GEPIA2 (http://gepia2021.cancer-pku.cn/ (accessed on 13 July 2025)) using the TCGA/GTEx expression data. * = *p* < 0.05.

**Figure 5:**
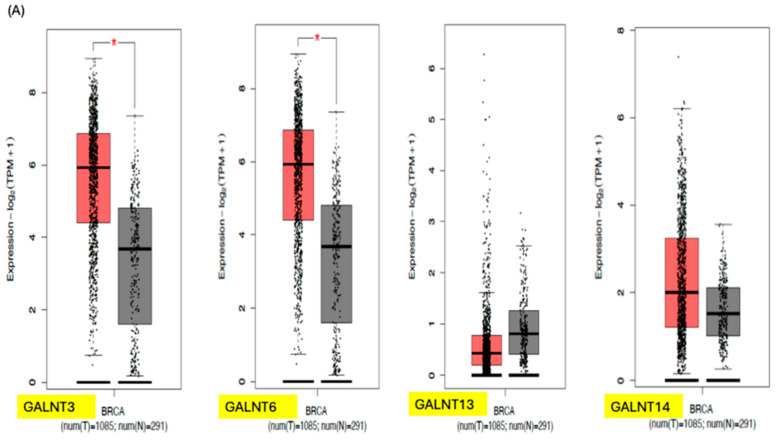

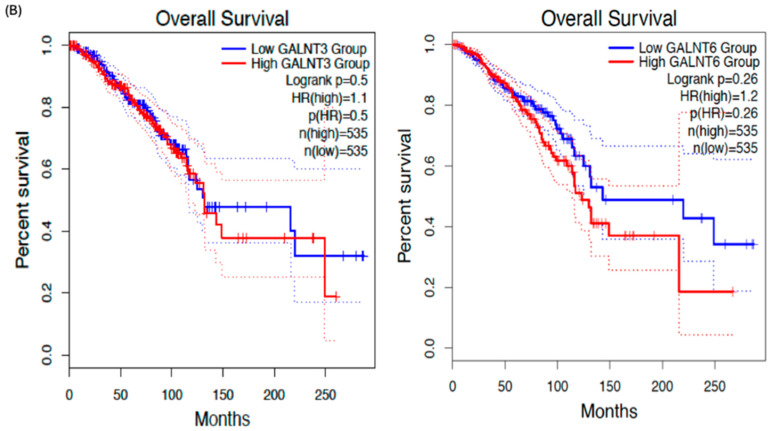
(**A**) Gene expression profile of the various GALNTs in breast cancer showing higher GALNT3 and 6 expressions in cancer cells compared to normal cells. (**B**) Kaplan–Meier survival curve based on GALNT expressions in breast cancer showing a slightly improved survival in cancers with low GALNT6 expression. Dotted lines indicate 95% confidence interval. The figures are extrapolated from GEPIA2 (http://gepia2021.cancer-pku.cn/ (accessed on 13 July 2025)) using the TCGA/GTEx expression data. * = *p* < 0.05.

Differential expression is associated with prognostic outcomes in various human cancers, mediated via the mechanisms described in Table 3. The gene expression levels of the various GALNTs in cancers compared with normal tissues were extrapolated from GEPIA2 (http://gepia2021.cancer-pku.cn/ (accessed on 13 July 2025)) and are shown in Figure 4 and Figure 5 [91]. GALNTs, therefore, may be used as biomarkers of prognosis and chemotherapeutic resistance. However, redundancy among isoforms complicates targeting due to functional overlap between isoforms and context-specific isoform switching, which makes it difficult for targeted therapies to be developed [92]. Our review emphasizes the fact that differential GALNT expression is associated with prognostic outcomes in various human cancers, mediated via the mechanisms described in Table 4 [93,94,95,96,97,98,99,100,101,102,103,104,105,106]. Nevertheless, further investigations are required to delineate the isoform-specific functional roles of individual GALNT family members, given their partially overlapping yet context-dependent enzymatic activities and substrate specificities. Comprehensive characterization at the transcriptional, post-transcriptional, and post-translational levels will be critical to uncover differential regulatory mechanisms and tissue- or disease-specific functions. Moreover, elucidating the molecular basis of intra-family compensatory dynamics, including potential transcriptional upregulation, altered substrate promiscuity, or subcellular delocalization in response to loss or inhibition of specific isoforms, will be essential for the rational design of targeted therapeutic interventions that can circumvent redundancy and achieve sustained glycosylation modulation in cancers.

## 4. Conclusions

GALNTs are key regulators of mucin-type *O*-glycosylation and exert diverse, isoform-specific effects on tumor progression, immune modulation, and metastasis. These enzymes are implicated in cancer progression through effects on proliferation, EMT, metastasis, and microenvironmental interactions. However, their roles remain limited by overlapping isoform functions, poorly defined substrates, and insufficient mechanistic clarity, especially regarding immune modulation. Current models often lack human-specific glycan features, and isoform-selective inhibitors are not yet clinically available. Diagnostic and therapeutic translation is still at an early stage. Future research should focus on mapping GALNT-specific substrates, developing selective inhibitors, characterizing glycan structures, and elucidating their roles in immune evasion. Integration of glycoproteomics with immuno-oncology may enable the creation of novel therapeutic strategies.

## Figures and Tables

**Table 1:** Expression of GALNTs in various human tissues (adapted from [4]).

GALNT	Human Tissues Expressed in	Family Subclassification	Chromosome Locus
T1	Widely distributed	Ia	18q12.1
T2	Widely distributed	Ib	1q41–q42
T3	Bone, sperm	Ic	2q24–q31
T4	Lung	IIa	12q21.33
T5	Stomach	Id	2q24.1
T6	Stomach, colon	Ic	12q13
T7	Sublingual gland	IIb	4q34.1
T8	Colon, testes	Ie	12p13.3
T9	Brain	Ie	12q24.33
T10	Kidney	IIb	5q33.2
T11	Prostate	If	7q36.1
T12	Colon	IIa	9q22.33
T13	Brain	Ia	2q24.1
T14	Kidney	Ib	2p23.1
T15	Placenta	Id	3p25.1
T16	Brain, heart	Ib	14q24.1
T17	Brain, ovary	IIb	4q34.1
T18	Widely distributed	Ie	11p15.3
T19	Brain	Ie	7q11.23
T20	Testes	If	7q36.1

**Table 2:** Some examples of the various GALNTs and their functional role in human cancers.

GALNT Isoform	Tissue Involved	Substrate or Pathway Involved	Functional Role	References
GALNT1 and -T2	Hepatocellular cancer	EGFR	Modifies the activity of EGFR; dysregulation leads to malignant behavior	[21,22,23]
GALNT3 and -T14	Ovarian cancer	MUC1; EGFR/mTOR	Increased activity leads to increased MUC1 expression, resulting in increased cell proliferation, cell migration, and invasion	[24,25,26]
GALNT4	Pancreatic cancer	MUC1	Induces *O*-GalNAc glycosylation of MUC1 to enhance MUC1 protein stability	[27]
GALNT6	Breast cancer	MUC1 & MUC4, α2-macroglobulin	Drives clustered *O*-GalNAc addition; activates PI3K/Akt signaling to increase invasion and tumor growth	[28,29]
GALNT7	Prostate cancer	*FOXO*1, androgen regulator	Upregulation affects immune activity and cell signaling	[30,31]

**Table 3:** The dual role of GALNTs as oncogenic and tumor suppressors in various cancers.

GALNT Isoform	Tumor Role	Mechanism	Primary Cancer	Source
GALNT1	Oncogenic	Glycosylates Notch to induce EMT and metastasis	Breast	[56]
GALNT2	Oncogenic	Activates Notch/Hes1-PI3K/ Akt axis	Lung	[77]
GALNT2	Suppressive	Maintains glycan profile; suppresses invasion	Gastric	[58]
GALNT3	Suppressive	Suppresses Wnt via TNFR1 and c-MET glycosylation	Lung	[89]
GALNT6	Oncogenic	Alters mucin-type *O*-glycosylation, causing Wnt activation	Colorectal	[87]
GALNT10	Suppressive	Cell surface glycoproteome remodeling, preventing EMT and migration	Ovarian	[90]
GALNT14	Oncogenic	Enhances β-catenin glycosylation, causing Wnt activation	Lung	[78]

**Table 4:** Prognostic impact of the various GALNTs on common human cancers.

Primary Cancer	GALNT Expression Pattern	Prognostic Impact	References
Breast (BRCA)	High GALNT6, -T13, -T14 in advanced disease.	GALNT6: ↑ associated with advanced nodal stage and poor prognosis. GALNT13: ↑ associated with tumor progression and metastasis. GALNT14: ↑ linked to lung-specific metastasis and reduced distant metastasis-free survival.	[28,88,93,94]
Colon	Low GALNT3, -T4, -T6, -T12 in advanced disease.	GALNT3: ↓ associated with lower survival rate; poor expression in poorly differentiated cancers. GALNT4: ↓ associated with increased metastatic potential and lower survival rate. GALNT6: ↓ associated with shorter survival; lower expression in BRAF-mutated and KRAS wild-type cancers. GALNT12: ↓ associated with shorter survival and metastasis.	[95,96,97,98]
Gastric	GALNT3, -T5, -T6, -T10 have varying impacts on outcomes	GALNT3: ↑ associated with good prognosis. GALNT5: ↓ associated with poorly differentiated gastric adenocarcinomas, advanced stage, lymph node metastasis, and poor prognosis. GALNT6: ↑ associated with correlation with advanced clinical stage, distant metastasis, and poor survival. GALNT10: ↑ associated with shorter survival, lymph node, and distant metastasis.	[99,100,101,102]
Pancreatic	GALNT3, -T5, -T6 have varying impacts on outcomes	GALNT3: ↓ contribute to dedifferentiation and increased aggressiveness during tumor progression. GALNT5: ↑ associated with poor survival. GALNT6: ↑ associated with favorable tumor biology and improved survival.	[103,104,105]
Thyroid	High GALNT3 expression in poor prognosis.	GALNT3: ↑ contribute to dedifferentiation and increased aggressiveness during tumor progression.	[106]

↑ represents increase in GALNT and ↓ represent decrease in GALNT.

## Data Availability

No new data were created or analyzed in this study.

## Abbreviations

The following abbreviations are used in this manuscript:

3D	Three-dimensional
AKT	Ak strain transforming
BMP	Bone morphogenetic protein
C1GALT1	Core 1 synthase, glycoprotein-N-acetylgalactosamine 3-beta-galactosyltransferase 1
CCL5	C-C motif chemokine ligand 5
CGN	Cis-Golgi network
c-Jun	Transcription factor Jun
COPI	Coat Protein I
COSMC	Core-1 β1-3galactosyltransferase-specific chaperone 1
CTL	Cytotoxic T lymphocytes
CTLA-4	Cytotoxic T-lymphocyte-associated protein 4
CRISPR	Clustered regularly interspaced short palindromic repeats
CXCL10	C-X-C motif chemokine ligand 10
EGF	Epithelial growth factor
EGFR	Epithelial growth factor receptors
EMT	Epithelial-mesenchymal transition
ER	Endoplasmic reticulum
ErbB	Erythroblastic oncogene B
ERK8	Extracellular Signal-Regulated Kinase 8
FOXO1	Forkhead box O1 transcription factor
FUT	Fucosyltransferase
GALA	GALNT activation
GALNT	N-acetylgalactosaminyltransferase
GlcNAc	N-Acetylglucosamine
GMP-AMP	Guanosine monophosphate-adenosine monophosphate
GT	Glycosyltransferase
GT-A	Glycosyltransferase fold A
GT-B	Glycosyltransferase fold B
GT-C	Glycosyltransferase fold C
HCC	Hepatocellular carcinoma
Her2	Human epidermal growth factor receptor 2
KRAS	Kirsten ras oncogene homolog
LAG-3	Lymphocyte-activation gene 3
MMP	Matrix metalloproteinase-2
mTOR	mammalian target of rapamycin
MUC1	Mucin-1
NICD	Notch intracellular domain
onfFN	Oncofetal fibronectin
PD-L1	Programmed Cell Death Ligand 1
PI3K	Phosphoinositide 3-kinase
PTEN	Phosphatase and tensin homolog
PTM	Post-translational modification
Ser	Serine
siRNA	Small interfering RNA
SLUG	SNAI2 gene
SNAIL	Zinc finger protein SNAI1
ST3Gal	ST3 beta-galactoside alpha-2,3-sialyltransferase 1
STING	Stimulator of interferon genes
TGF-β	Transforming growth factor beta
Thr	Threonine
Tn	Thomsen-nouveau
TGN	Trans-Golgi network
TME	Tumour microenvironment
UBE2C	Ubiquitin-conjugating enzyme E2C
UDP-GalNAc	Uridine diphosphate N-acetylgalactosamine
VEGF	Vascular endothelial growth factor
VTC	Vesicular tubular clusters
VVA	Vicia villosa Lectin
ZEB1	Zinc finger E-box binding homeobox 1
